# Expert-approved best practice recommendations on the use of sedative drugs and intentional sedation in specialist palliative care (SedPall)

**DOI:** 10.1186/s12904-023-01243-z

**Published:** 2023-09-04

**Authors:** Christoph Ostgathe, Claudia Bausewein, Eva Schildmann, Jeremias Bazata, Violet Handtke, Maria Heckel, Carsten Klein, Alexander Kremling, Sandra Kurkowski, Sophie Meesters, Andreas Seifert, Jorge Luis Torres Cavazos, Kerstin Ziegler, Christian Jäger, Jan Schildmann

**Affiliations:** 1 Department of Palliative Medicine, CCC Erlangen – EMN, Universitätsklinikum Erlangen, Friedrich- Alexander-Universität Erlangen-Nürnberg (FAU), Werner-von-Siemens-Straße 34, Erlangen, 91052 Germany; 2 Department of Palliative Medicine, LMU University Hospital, LMU Munich, Marchioninistraße 15, 81377 Munich, Germany; 3 Interdisciplinary Centre for Palliative Medicine, Medical Faculty, University of Augsburg, Stenglinstraße 2, Augsburg, 86156 Germany; 4 Institute for History and Ethics of Medicine, Interdisciplinary Center for Health Sciences, Martin Luther University Halle-Wittenberg, Magdeburger Str. 8, 06112 Halle (Saale), Germany; 5 Paderborn Centre for Educational Research and Teacher Education – PLAZ Professional School, Paderborn University, Warburger Str. 100, 33098 Paderborn, Germany; 6 Department of Criminal Law, Criminal Procedural Law, Commercial Criminal Law and Medical Criminal Law, Friedrich-Alexander-Universität Erlangen-Nürnberg (FAU), Schillerstraße 1, 91054 Erlangen, Germany

**Keywords:** Palliative care, Terminal care, Hypnotics and sedatives, Practice guidelines, Empirical research, Community-based participatory research

## Abstract

**Background:**

The use of sedative drugs and intentional sedation in end-of-life care is associated with clinical, ethical and legal challenges. In view of these and of the issue’s great importance to patients undergoing intolerable suffering, we conducted a project titled SedPall (“From anxiolysis to deep continuous sedation – Development of recommendations for sedation in palliative care“) with the purpose of developing best practice recommendations on the use of sedative drugs and intentional sedation in specialist palliative care and obtaining feedback and approval from experts in this area.

**Design:**

Our stepwise approach entailed drafting the recommendations, obtaining expert feedback, conducting a single-round Delphi study, and convening a consensus conference. As an interdisciplinary group, we created a set of best practice recommendations based on previously published guidance and empirical and normative analysis, and drawing on feedback from experts, including patient representatives and of public involvement participants. We set the required agreement rate for approval at the single-round Delphi and the consensus conference at ≥80%.

**Results:**

Ten experts commented on the recommendations’ first draft. The Delphi panel comprised 50 experts and patient and public involvement participants, while 46 participants attended the consensus conference. In total, the participants in these stages of the process approved 66 recommendations, covering the topics “indications”, “intent/purpose [of sedation]”, “decision-making”, “information and consent”, “medication and type of sedation”, “monitoring”, “management of fluids and nutrition”, “continuing other measures”, “support for relatives”, and “team support”. The recommendations include suggestions on terminology and comments on legal issues.

**Conclusion:**

Further research will be required for evaluating the feasibility of the recommendations’ implementation and their effectiveness. The recommendations and the suggested terminology may serve as a resource for healthcare professionals in Germany on the use of sedative drugs and intentional sedation in specialist palliative care and may contribute to discussion on the topic at an international level.

**Trial Registration:**

DRKS00015047 (German Clinical Trials Register)

**Supplementary Information:**

The online version contains supplementary material available at 10.1186/s12904-023-01243-z.

## Background

Sedative drugs are widely used for symptom control in palliative care, for purposes including the relief of agitation or anxiety. Due to their ability to induce a sustained reduction in consciousness, their use entails complex ethical, legal and cultural challenges [1]. A reduction in consciousness, while it may be consistent with the patient’s wishes, may lead to an unwanted loss of autonomy and a decreased ability to communicate with healthcare professionals and family members [2]. The current debate in this area largely focuses on what is generally termed “palliative sedation”, defined by the European Association for Palliative Care (EAPC) as “the monitored use of medications intended to induce a state of decreased or absent awareness (unconsciousness) in order to relieve the burden of otherwise intractable suffering in a manner that is ethically acceptable to the patient, family and health-care providers” [3]. The umbrella term “palliative sedation” encompasses various possible practices differing in terms of the depth (light/deep) or the duration of sedation (temporary/continuous). Sedation as a side-effect of medication (secondary sedation) [4], albeit not covered by this definition, unfolds a similar impact on patients’ autonomy and capacity to communicate. The current international literature demonstrates the challenges facing this area of medicine by reporting a variety of indications for “palliative sedation” [3, 5, 6] and the use of a number of different medications and dosages. International guidance exists, but its terminology and content are heterogeneous [2, 7–11]. In Germany, physicians and teams might adhere to the German translation of the EAPC recommendations for clinical practice, the guidelines published by the Comprehensive Cancer Centres in Germany [12], or the recommendations of the German Academy for Ethics in Medicine [13], as far as they are aware of them [14]. The lack of precise and generally accepted terminology and of a consistent framework impedes transparency in describing sedation practices and the evaluation of the associated clinical and ethico-legal challenges [7, 15, 16]. The authors of the present paper have recently suggested the term “intentional sedation” to indicate a deliberate decision to reduce the patient’s consciousness. Intentional sedation is the “result or process of sedating a patient as a means of achieving a previously defined treatment goal”, for example in cases of intolerable and otherwise untreatable suffering [17]. The terminology in this area also distinguishes temporary sedation (the patient is sedated only for a certain period of time) from sedation until death (the patient remains continuously under sedation until she or he dies).

The purpose of this study was to develop and gain consensus on best practice recommendations for the use of sedative drugs in specialist inpatient and home palliative care, spanning the spectrum from symptom control to intentional sedation for the relief of intolerable and otherwise untreatable suffering. Outside the specialist context, other recommendations may apply, due principally to limited availability of resources; such recommendations are not the subject of this study.

## Methods

### Study design

We carried out a multi-stage process that drew up and attained consensus on best practice recommendations on the use of sedative drugs and intentional sedation in specialist palliative care.

We outline the development of the empirical recommendations in accordance with the CREDES Guidance on Conducting and Reporting Delphi Studies in palliative care [18]. We report patient and public involvement throughout the procedure in line with the GRIPP2 reporting checklists [19].

### The project

The project, titled “From anxiolysis to deep continuous sedation – Development of recommendations for sedation in palliative care (SedPall)“ (funded by the German Federal Ministry of Education and Research, BMBF 01GY1702A-C) ran from 2017 to 2021. It was conducted by a multidisciplinary consortium comprising experts from the fields of ethics, gerontology, law, nursing science, palliative care/medicine, philosophy, and sociology, from four institutions (Department of Palliative Medicine, LMU University Hospital, LMU Munich; Department of Palliative Medicine, Universitätsklinikum Erlangen; Institute for History and Ethics of Medicine, Interdisciplinary Center of Health Sciences, Martin Luther University Halle-Wittenberg; and Department of Criminal Law, Criminal Procedural Law, Commercial Criminal Law and Medical Criminal Law, Friedrich-Alexander-Universität Erlangen-Nürnberg (FAU)). Among the previous work by members of the consortium that informed the development of the recommendations were systematic reviews of published guidance on sedation; empirical data on views and clinical practices around the use of sedative drugs in specialist palliative care, collected in cooperating specialist palliative care services (we term these services our “project partners”); and normative considerations on legal and ethical aspects of the issue [7–9, 20].

Patient and public involvement (PPI) participants provided continuous support to the project, enabling us to take patients’ and families’ views on board and obtain their advice on aspects of the issue with particular relevance to them. The PPI participants are experts in the sense of individuals with lived experience of situations in which intentional sedation was discussed or used for members of their families who received specialist palliative care in one of the consortium’s university hospitals.

The scientific advisory board to the project consisted of 21 national and international palliative care experts with a background in medicine, nursing, ethics, law, and/or psychology of whom six were non-German speakers. Some of them were seconded to the project from the German Association for Palliative Medicine (DGP).

**Fig. 1:**
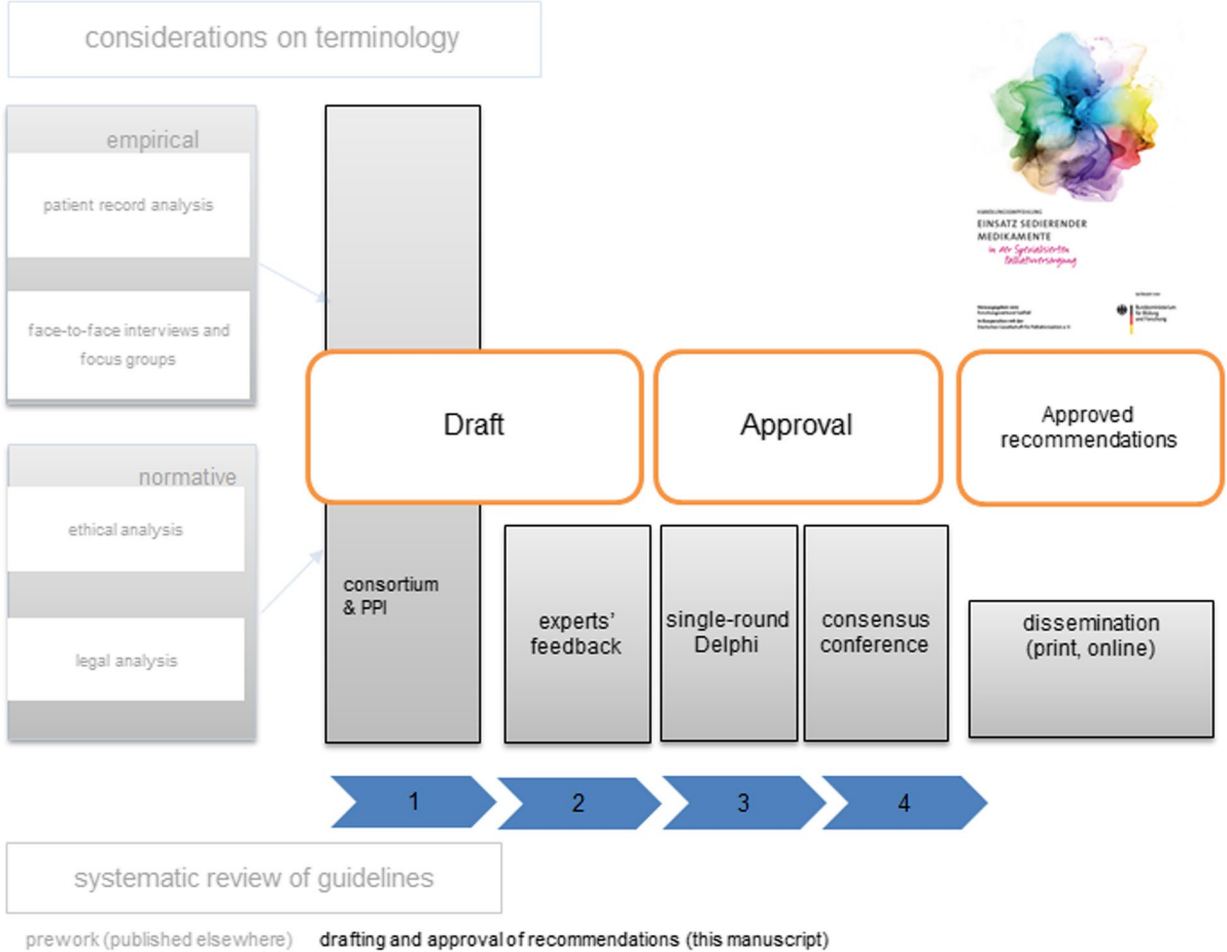
Study design; PPI = patient and public involvement

The recommendations achieved final approval in a single-round Delphi study and a consensus conference (see Fig. 1).

### Our stepwise approach

The drafting and approval of the recommendations comprised four steps:

#### Step 1: Drafting recommendations

Four sub-projects conducted preparatory work focusing on various aspects of the use of sedatives and intentional sedation at the end of life (see German Clinical Trials Register DRKS00015047). Sub-project 1 (clinical centre) investigated clinical practices around the use of sedative drugs in palliative care units and home care settings, using chart reviews of patient records [21–23]. Sub-project 2 (clinical centre) conducted face-to-face interviews and focus groups exploring views held by patients, relatives, and staff on intentional sedation at the end of life [14]. Sub-project 3 (medical ethics) analysed ethical aspects of the issues and refined the terminology [7]. Sub-project 4 (medical law) analysed the legal aspects of the issues. At a joint meeting, consortium members communicated key findings of their sub-projects and defined and discussed overarching themes that occurred across the sub-projects. Sub-projects 1 and 2 drafted recommendations structured by topic, on the basis of systematic reviews of existing guidance on sedation, the empirical data referenced above in the context of previous work, and the terminology developed during the work up to that point [7, 9, 17, 20]. Sub-projects 3 and 4 added an introductory section around terminology (on the interdisciplinary development of terminology in this area, see reference 7) and legal aspects of the issue. Consortium members subsequently held several video calls during which they discussed and amended the draft, and eventually approved its initial version.

#### Step 2: Expert feedback on initial draft of recommendations

We invited feedback from German-speaking experts from the project’s scientific advisory board with clinical experience in the use of sedative drugs and intentional sedation in palliative care and/or track records of academic work on the topic in medicine, nursing, law, and/or ethics and from project partners. The experts commented on the recommendations’ initial version in general and/or on individual recommendations. The consortium members and the PPI participants discussed this feedback in a videoconference. The group adapted the recommendations in accordance with the feedback; where it rejected one of the proposed recommendations, it documented the reasons for this directly in the draft worked on during the videoconference (live documentation) [24]. The consortium then gave final approval to the revised recommendations.

#### Step 3: Single-round Delphi study

The next step consisted in a single-round Delphi study, for which we consciously brought together a panel of experts from a range of professional backgrounds. Inclusion criteria were clinical expertise in the use of sedative drugs or research activities in inpatient or home-based palliative care. The panel included all members of the consortium. PPI participants and project partners from the SedPall project also took part. The participants indicated their approval or non-approval of each recommendation (yes/no). Consensus was defined at an agreement rate of ≥ 80% among all participants, on the basis of percentage agreement rates used in previous studies [18, 25].

#### Step 4: Consensus conference

A consensus conference took place for the purpose of discussing and approving the recommendations that did not achieve consensus in the single-round Delphi (< 80% approval). The Delphi panel took part in the conference. Participants worked together to adapt non-approved recommendations via livedocumentation [24] and approved them via live voting (with a yes or no to approval). We used the Zoom polling feature for live voting.

### Ethics committee approval

The overall study was approved by the Local Research Ethics Committes of Friedrich- Alexander-Universität Erlangen-Nürnberg (FAU) (No. 376_16 Bc, 22 December 2016) and Ludwig-Maximilians-Universität München: (No. 18–191, 19 April 2018) and by the appointed data protection officers. Participants provided written informed consent.

## Results

### Draft recommendations

Initially, the consortium drafted 74 recommendations on the ten predefined themes of “indications”, “intent/purpose [of sedation]”, “decision-making”, “information and consent”, “medication and types of sedation”, “monitoring”, “management of fluids and nutrition”, “continuation of other measures”, “support for relatives”, and “team support”. For each recommendation, the recommendations’ authors indicated whether it applied to inpatient or home care settings or both. The introductory section included information on ethico-legal issues and terminology.

### Expert feedback

Ten out of 28 German-speaking advisory board members and project partners provided feedback and comments on the first draft. Nine of these individuals were male; eight were physicians (*n* = 3 specialists in internal medicine, *n* = 2 general practitioners, *n* = 3 anaesthesiologists), one was a medical ethicist and one was a nurse; all had long-standing experience in palliative care. They provided 73 comments in total.

Consortium members, and PPI participants who wished to engage in this process, revised the draft in accordance with the summarised comments at four videoconferences. They adapted eleven recommendations and struck out a further eight, incorporating relevant aspects of some of those removed into other recommendations. A small number of recommendations initially had separate versions for each setting; the working group subsequently merged each of these into one recommendation covering both settings; this was the case primarily for recommendations relating to the decision-making process. The descriptive nature of two recommendations (relating to the impact of sedation on relatives and team members and to raising treatment teams’ awareness of this impact) led to their inclusion in the section detailing the background to the recommendations and not as recommendations in their own right. The group adjusted recommendations on existential suffering and intentional sedation that had been the subject of contentious discussion. A further addition on indications for sedation clarified the point that the patient’s desire for sedation does not constitute an indication for intentional sedation, but should result in an assessment of whether intentional sedation is indicated. The group added a rationale for the recommendation to measure vital signs in special situations to avoid shortening life. A further clarification stated that the team may only inform relatives of planned sedation if this is in accordance with the patient’s wishes.

The 66 recommendations that remained after this process were put to the subsequent single-round Delphi panel. In addition, the consortium revised the introduction and its sections on terminology and legal aspects in light of the experts’ feedback.

### Single-round Delphi study

In January 2021, we invited 46 German-speaking experts (including 15 consortium members) and nine PPI participants to take part in a single-round Delphi in the form of an online survey developed for this study, subsequently sending one reminder. A total of 50 individuals (44 experts and 7 patient and public representatives) took part (participation rate: 90.9%). Most participants were aged 50 years or older. Most of the professional participants were physicians and had more than 10 years of experience in patient care (see characteristics of participants in Table 1).

**Table 1:** Single-round Delphi: participants’ characteristics (*n* = 50)

Age group	n/a	3 (6%)
	20–29 years	3 (6%)
	30–39 years	5 (10%)
	40–49 years	9 (18%)
	50–59 years	17 (34%)
	60–69 years	7 (14%)
	70–79 years	4 (8%)
	80–89 years	2 (4%)
Gender	male	26 (52%)
	female	24 (48%)
Professional background	physician	26 (52%)
	patient and public involvement participant	7 (14%)
	specialist in ethics/philosophy/theology	5 (10%)
	legal expert	3 (6%)
	nurse	2 (4%)
	psychologist	2 (4%)
	social worker	2 (4%)
	sociologist	2 (4%)
	public health specialist	1 (2%)
Years of experience in patient care	n/a	12 (24%)
	1 to 5	9 (18%)
	6 to 10	4 (8%)
	11 to 15	12 (24%)
	16 to 20	5 (10%)
	> 20	8 (16%)
Setting	inpatient	24 (48%)
	home care	17 (34%)
	n/a	9 (18%)

Of 66 recommendations, two did not achieve ≥ 80% consensus. Fifty-two recommendations attained an approval rate of ≥ 90%.

### Consensus conference

Forty-six participants attended the online consensus conference (consortium members: *n* = 12, scientific advisory board members: *n* = 9, experts from German Association for Palliative Medicine: *n* = 3, patient and public representatives: *n* = 5, project partners from inpatient and home care settings: *n* = 17), which took place in February 2021. The conference achieved consensus on two remaining recommendations after a shared process of adaptation by the consensus conference participants. Both remaining recommendations thus attained the necessary approval rate of ≥ 80% in the live voting.

The total of 66 recommendations finally approved cover ten themes, as shown in Table 2.

**Table 2:** Approved recommendations

Themes		Recommendations	setting
Indication	1	Before sedating medication is used, the indication must be defined and documented.	SIPC, SPHC
	2	Sedating medication can be used to relieve symptoms that patients find distressing, such as anxiety and agitation, without intending to alter consciousness.	SIPC, SPHC
	3	Sedating medication can be used to relieve insomnia, if experienced as distressing by the patient. In this context, a temporary and reversible change in consciousness is intended (RASS-PAL < 0)^a^.	SIPC, SPHC
	4	Sedating medication can be administered to prevent suffering during or upon termination of medical measures.	SIPC, SPHC
	5	In the case of distressing symptoms which, despite all proportionate measures to relieve them (measures administered on expert level), have not been sufficiently alleviated and remain unbearable for the patient, intentional sedation is indicated.	SIPC, SPHC
	6	In medical crisis situations, such as acute haemorrhage or acute obstruction of the respiratory tract, in addition to opioid treatment of possible dyspnoea, intentional - if necessary deep - sedation is indicated.	SIPC, SPHC
	7	Existential suffering is not an indication for deep continuous sedation until death without prior temporary sedation.	SIPC, SPHC
	8	In general, the maintenance of deep sedation until death is only indicated when it can be assumed - with almost complete certainty - that a reduction in the level of sedation would lead to unbearable suffering again.	SIPC, SPHC
	9	The wish to die is not an indication for the administration of potentially sedating medication and therefore also not for intentional sedation.	SIPC, SPHC
	10	The desire for sedation should result in an assessment whether intentional sedation is indicated.	SIPC, SPHC
Intent/ Purpose	11	Sedating medication should be administered for the purpose of relieving symptoms, relieving suffering, or preventing imminent suffering during or upon termination of medical measures.	SIPC, SPHC
	12	Before and during intentional sedation, the team ensures that the suffering of the patient remains the central focus and that the sedating medication is not used for the purpose of reducing the burden on the family or the team.	SIPC, SPHC
	13	Intentional sedation must not be administered to hasten the death of the patient.	SIPC, SPHC
	14	Intentional sedation which results in a limitation of mobility, may (only) be administered without judicial authorisation if the prevention of leaving the place of residence is not the primary purpose but a side effect of the primary intended relief of suffering.	SIPC, SPHC
Decision-making	15	The decision to use intentional sedation will be made in accordance with the (presumed) will of the patient.	SIPC, SPHC
	16	Before intentional sedation, the patient or their legal representative and the treatment team must determine who is involved in the decision-making process.	SIPC, SPHC
	17	In the case of diseases in which severe respiratory distress and/or a haemorrhage can be expected (e.g. tumours of the head or neck, motor neurone disease, COPD, pulmonary fibrosis), the option of symptom-relieving intentional sedation should be discussed in advance with the patient or their legal representative. This conversation should be documented in the patient’s record or health care planning documentation for the last phase of life.	SIPC, SPHC
	18	The assessment of whether symptoms remain refractory and unbearable for the patient, despite all proportionate (expert delivered) measures to relieve symptoms, takes place during a multi-professional case conference. In cases of existential suffering, psychological and pastoral competencies should be included in the case conference.	SIPC, SPHC
	19	In cases of ethical conflict, the decision-making process relating to whether or not intentional sedation is to be administered should be supported by ethics counselling/an ethics case conference. Ethics counselling/ethics case conferences must be transparently documented in the patient’s record.	SIPC, SPHC
	20	If intentional sedation is initiated during acute episodes of symptom exacerbation, when multi-professional discussion of the case is not possible, then this must be retrospectively carried out as soon as possible to confirm or revise the course of treatment.	SIPC, SPHC
	21	If the use of a medication results in an unwanted reduction in consciousness, then an adjustment to the medication (dose, substance) to reverse the reduction in consciousness is to be considered or a decision must be made promptly at a case conference as to whether intentional sedation is indicated and corresponds to the (presumed) will of the patient. Only then intentional sedation - using suitable medication - is deemed appropriate.	SIPC, SPHC
	22	The decision-making process for intentional sedation, the parties involved in the decision-making process, and the results of the decisions must be transparently documented in the patient’s record.	SIPC, SPHC
Information/Consent	**Preliminary remark**	Consent must be given by the patient. If the patient is unable to provide consent, a legal representative should be consulted to determine the will of the patient.	
	23	Before intentional sedation, the patient or their legal representative will be informed of all relevant indications, intentions, effects, planned duration, adverse effects, risks, potential effects on length of life (both in regard to shortening or prolongation), possible course without sedation, and voluntary nature of consent to the sedation.	SIPC, SPHC
	24	When using medication that is not specifically used for sedation but may cause sedation as a side effect, the patient or their legal representative will be informed of this risk.	SIPC, SPHC
	25	The treatment team must involve the patient’s relatives in the process of providing information on the intentional sedation if this is the wish of the patient or their legal representative.	SIPC, SPHC
	26	The patient, and with the patient’s consent, their relatives are to be informed that the patient’s ability to communicate during the use of sedating medication will be limited, especially in cases of intentional sedation. If the patient no longer possesses the capacity to consent, the legal representative of the patient should receive the necessary information.	SIPC, SPHC
	27	To ensure the patient’s right to self-determination, after providing the relevant information and a suitable time window, the patient will be asked to consent to administration of intentional sedation (informed consent). If the patient no longer possesses the capacity to consent, the legal representative of the patient should be asked to provide the necessary consent.	SIPC, SPHC
	28	Before the administration of intentional sedation, decisions to be made during the period of (potential) incapacity to consent should be discussed with the patient (if the patient is unable to consent, then with the patient’s legal representative). The discussion covers aspects such as rituals, nursing measures, duration of sedation, targeted level of sedation, possible attempts to awaken the patient (including the possible foregoing of the same), the management of other medications, and (artificial) hydration and nutrition.	SIPC, SPHC
	29	If intentional sedation is initiated during acute episodes of symptom exacerbation, and it is not possible to provide the necessary information, this should be provided as soon as possible, if necessary, by retrospectively informing the patient’s legal representative.	SIPC, SPHC
	30	The information process and the type of information provided are to be transparently documented in the patient’s record.	SIPC, SPHC
Medication and types of sedation	31	When using sedating medication, the substance selection is based on the indication, intention, effect, and duration of the treatment and possible adverse effects.	SIPC, SPHC
	32	Intentional sedation uses the lowest possible dose of the medication to achieve the level of sedation necessary to relieve the patient’s suffering. Therefore, the dose should always ensure that the patient’s suffering is reduced to a level tolerable for the patient and that the sedation level is no deeper than necessary.	SIPC, SPHC
	33	Generally, on initiation a medication dose is chosen to achieve light to moderate sedation (RASS-PAL − 1 to -2) [26]. Subsequently, the dose is adjusted in accordance with the recommendation in 2).	SIPC, SPHC
	34	In case of acute crisis (e.g. acute respiratory tract obstruction, severe haemorrhage), an initial medication dose to achieve a deep level of sedation (RASS-PAL ≤ -3)^a^ can be selected.	SIPC, SPHC
	35	In the event of changes in respiratory activity (bradypnea, hypoventilation) during intentional sedation, it should be critically assessed whether these changes are due to the dying phase or the medication dose. If the medication dose is found to be the cause of the change in respiration, then a dose reduction adapted to the relief of suffering should be considered. If the reduction in respiratory activity is due to the dying phase, then no dose reduction is necessary.	SIPC, SPHC
	36	Intentional sedation should initially be administered as temporary sedation and then re-evaluated after a predefined time period.	SIPC, SPHC
	37	Intentional sedation in case of existential suffering is initially administered as temporary sedation for a predefined time period (up to a maximum of 24 h).	SIPC, SPHC
	38	Benzodiazepines, e.g. midazolam, are suitable for intentional sedation. Generally, these medications are the first choice, especially for patients requiring a reduction in anxiety levels and/or anti-epileptic effects. In the case of delirium, they should only be administered in combination with antipsychotic medication.	SIPC, SPHC
	39	Antipsychotics with sedating (secondary) effects, e.g. levomepromazine, are a suitable second choice medication for intentional sedation. They can be administered in combination with benzodiazepines in cases in which benzodiazepines alone are inadequate to achieve sufficient relief of suffering.	SIPC, SPHC
	40	Propofol is suitable for intentional sedation in cases in which other types of medication have not resulted in sufficient relief of suffering.	SIPC
	41	Propofol is not suitable for intentional sedation in the home care setting.	SPHC
	42	Opioids are not suitable for use in intentional sedation. Increasing the dose of an existing opioid therapy is also not a suitable means of intentional sedation. During intentional sedation, opioid treatment to reduced pain levels and/or treat dyspnoea is continued and the dose is adjusted as needed to ensure relief of pain and/or dyspnoea.	SIPC, SPHC
Monitoring	43	During sedation, the situation is re-evaluated by the person administering treatment and the dose adjusted to ensure the suffering is relieved to an acceptable level and that the level of sedation is no more than that required to relief the suffering.	SIPC, SPHC
	44	The criteria for regular re-evaluation of the overall situation are intensity of suffering (most important criterion), level of sedation, and adverse effects.	SIPC, SPHC
	45	The person administering intentional sedation is expected to use the patient’s relatives as an important supplementary source of information during regular re-evaluation.	SIPC, SPHC
	46	During intentional sedation, depending on the illness situation and the treatment goals, selected vital signs (e.g. respiratory rate, oxygen saturation, heart rate, and blood pressure) could additionally be monitored to ensure a stable clinical status of the patient within the framework of the agreed objectives and limits of treatment. Threshold values and corresponding consequences and reactions must be defined for monitored vital signs.	SIPC, SPHC
	47	During deep sedation outside of the dying phase, appropriate (vital) signs and parameters should be monitored to ensure that shortening of life is avoided as far as possible.	SIPC, SPHC
	48	The frequency of re-evaluation should be determined (and adjusted, as necessary) by the physician responsible for the intentional sedation, taking into consideration the planned type of sedation and the pharmacokinetic properties of the sedating medication. Differences between titration phase and maintenance phases have to be considered.	SIPC, SPHC
	49	As far as possible, the intensity of suffering should be assessed by directly asking the patient or their relatives, as well as by clinical observation (e.g. facial expression, sounds like groaning and screaming, body language, movements, agitation, tachycardia, and sweating).	SIPC, SPHC
	50	The depth intentional sedation is assessed based on reactions to being addressed and light, non-painful touching e.g. using RASS-PAL [26].	SIPC, SPHC
	51	The results of the re-evaluation of intentional sedation and the resulting consequences must be transparently documented in the patient’s record.	SIPC, SPHC
Management of fluids and nutrition	52	The decision to administer artificial hydration and/or nutrition must be made before or during sedation if the patient will no longer be able to eat and drink sufficiently on their own.	SIPC, SPHC
	53	The decision to determine whether the artificial administration of fluids and/or nutrition is indicated must be made separately from the decision on intentional sedation.	SIPC, SPHC
	54	In the case of intentional sedation, any decision on artificial hydration and/or nutrition is made with the patient or the patient´s legal representative or based on the presumed will of the patient and taking into consideration possible advantages and burdens as a result of these measures with regard to the treatment goals (relief of suffering).	SIPC, SPHC
	55	The decision relating to artificial hydration and/or nutrition during intentional sedation should be transparently documented in the patient’s record.	SIPC, SPHC
Continuing other measures	56	During intentional sedation, the patient will continue to be treated in the same dignified manner as before sedation. This includes addressing the patient (also in phases during which the patient is not conscious), announcing in advance actions that involve touching the patient, and adapting the surroundings to the given situation and, if necessary, in accordance with the previously discussed wishes of the patient.	SIPC, SPHC
	57	All nursing and medical measures are to be regularly evaluated and orientated towards the well-being of the patient. The measures should be adjusted to the changing conditions during intentional sedation and in accordance with the stated or presumed will of the patient.	SIPC, SPHC
	58	Measures to ensure symptom relief and patient well-being that were implemented before the intentional sedation are normally continued, regularly re-evaluated, and adjusted if necessary.	SIPC, SPHC
Support for relatives	59	With the consent of the patient, the relatives should be included from the beginning in the decision-making process related to intentional sedation.	SIPC, SPHC
	60	With the consent of the patient, relatives will be regularly informed of the patient’s current clinical situation and the expected course throughout the intentional sedation.	SIPC, SPHC
	61	The team offers support to the relatives regarding their emotional or spiritual needs resulting from the intentional sedation.	SIPC, SPHC
	62	The relatives are advised and, if necessary, instructed on how to support the patient during the intentional sedation and remain close to them, e.g. by talking, touching, creating a comforting atmosphere for the patient (e.g. favourite music, smells, singing well-known songs, reading aloud) and, if desired - are involved in the nursing care (e.g. mouth care).	SIPC, SPHC
	63	Before deep sedation, which is expected to continue until death, or sedation which may become deep continuous sedation, the patient and their relatives should be given the opportunity to say goodbye to one another if the situation allows it.	SIPC, SPHC
	64	After the death of the patient, the relatives will be given the opportunity to talk to members of the treatment team to discuss any remaining doubts concerning the intentional sedation.	SIPC, SPHC
Team support	65	All team members must fully understand the indications and treatment objectives of intentional sedation. The necessary discussions can take place at team meetings or during case conferences.	SIPC, SPHC
	66	The discussion of stressful situations relating to intentional sedation, e.g. a retrospective case review or conference, is recommended. The aim of these meetings is to discuss the factual and emotional challenges, help the team process stress, and continuously improve the care provided.	SIPC, SPHC

*SIPC *Specialist Inpatient Palliative Care, *SPHC *Specialist Palliative Home Care; the original recommendations, definitions and accompanying texts were professionally translated. This translation was revised and adapted to ensure internal consistency with the German version and finally agreed again within the SedPall consortium

Bush et al. 2014 [26]

Table 2: Recommendations approved by the end of the process; Legend: SIPC = specialist inpatient palliative care; SPHC = specialist palliative home care. A professional translation was made of the original recommendations, definitions and accompanying text; this translation was revised and adapted to ensure internal consistency with the German version within the SedPall consortium. RASS-PALL according to Bush (2014) [26].

### Dissemination

We disseminated the project’s results via a public closing conference, held online in April 2021, at which the consortium gave a presentation of the sub-projects’ key findings and shared excerpts from the recommendations. The 115 attendees comprised consortium members, representatives of project partners, scientific advisory board members and members of the public. Publication took place in print form (in German) and online (German and English) on the website of the German Association for Palliative Medicine (DGP) in the interests of providing open access to the recommendations and to raise awareness of them among those interested in the issue, including institutions and organisations providing palliative care [27, 28].

## Discussion

### Key results

This paper outlines the process of drawing up recommendations for the use of sedative drugs and intentional sedation in specialised palliative care, covering the spectrum of purposes from symptom control to deep continuous sedation and taking clinical, ethical and legal issues into account. Previously published evidence in this area could offer little guidance to the consortium due to the heterogeneity characterising definitions of key terms in much of this work [7, 15, 16]. We were unable to draw on the recently published preprint of the updated EAPC framework on palliative sedation, as it was not yet available during our work [29]. Preparatory normative and empirical analysis and the analysis of previously published guidance informed the development of the recommendations, which interdisciplinary experts with a clinical background in specialist palliative care and research, alongside PPI participants, subsequently approved [27]. Although developed for the German context, the recommendations could serve as an example on which other countries and healthcare systems might draw, with appropriate adaptations to the relevant national legal and clinical frameworks.

One major challenge in the use of sedative drugs at the end of life is the distinction between therapeutic administration of drugs of which a reduction in consciousness is a side effect, or secondary drug reactions, and intentional sedation [30–32]. Our recommendations seek to meet this challenge by carefully evaluating the nature and impact of any reduction in consciousness affecting the individual patient. If medication may have caused the reduction in consciousness, those treating the patient should consider adjusting the medication. Alternatively, it may be necessary to take a decision on whether intentional sedation is indicated, where distressing symptoms remain intolerable to the patient despite all proportionate measures to relieve them.

It is our hope that the recommendations will provide the best possible support to palliative care professionals in treating and caring for their patients in accordance with the law and in line with the current state of research. The recommendations should increase professionals’ self-confidence in the use of sedative drugs and support the process of multiprofessional team decision-making. It is our hope that they will promote standardisation in this area without limiting individualised care for patients [33]. Future research should seek to establish the feasibility of the recommendations’ implementation in specialist palliative care settings and the extent to which they improve professionals’ confidence in this area and the quality of care.

### Intentional sedation and generalist palliative care

The recommendations are intended for specialist palliative care settings. This restriction may appear to exclude from their applicability the majority of patients in end-of-life care, who receive generalist palliative care provided by general practitioners, home care services, staff in long-term care facilities, and staff on hospital wards. The recommendations may also be useful to primary care providers, which may, however, struggle to implement them fully due to limitations on resources such as multi-professional teams and out-of-hours service provision [20, 32]. In line with general recommendations on timely integrating palliative care in the treatment of for terminally ill patients, we consider it crucial to involve palliative care specialists at an early stage of treatment, where symptom control proves challenging and especially where a reduction of consciousness is the only means of achieving relief of symptoms [34]. A future project will centre on adapting the recommendations to generalist palliative care, taking into account the associated challenges [32, 35].

### Intentional sedation and specialist palliative home care

The recommendations focus on specialist palliative inpatient and home care. The year 1990 saw the first ever publication on sedation in palliative care that discussed patients treated with sedative drugs at home [36]. This work initiated an ongoing debate around which types of intentional sedation are possible in the home setting; previously published research and recommendations suggest that this depends on characteristics of the specific setting and particularly on the staffing levels specialist teams can provide [13, 20, 37, 38]. The recommendations we outline here are suitable for use in both inpatient and home care settings. Only propofol, a narcotic, is not recommended for use in home care because of its smaller therapeutic window and its higher risks compared to midazolam. This is in line with other national and regional recommendations [2, 38–40].

### Strengths and weaknesses of the study

The study’s interdisciplinary, holistic approach was among its major strengths, balancing the view from clinical practice with normative dimensions of the issues by virtue of the in-depth discussions that led to agreement on a shared terminology. We accounted for the fact that German law reserves the prescription of sedative drugs to physicians by including a relatively high proportion of physicians in the panel.

We faced a number of challenges during the study, among which was a necessary switch from face-to-face formats to videoconferences due to the COVID-19 pandemic. This had the potential to impact the quality of discussions; participants, however, rapidly became accustomed to the online formats, which indeed proved helpful to their attendance.

Our inclusion of consortium members in the Delphi panel may appear at first glance to have impacted its objectivity; we mitigated this potential limitation by the anonymity of the Delphi process, which was not a feature of the consortium members’ discussions.

It is possible to take the view that our use of only one Delphi round limited the quality of the consensus process. The iterative procedure in which the multidisciplinary and multiprofessional consortium created the first and consecutive drafts of the recommendations was driven by expert feedback and undergirded by the consortium members’ broad research and practical expertise in this area. In our view, this justifies the use of a single Delphi round.

The PPI participants had a consultative role in the process, meaning their opportunities to influence decisions such as those on methodology were limited. Nevertheless, representatives of patients and the public were involved in the final approval of the recommendations and had full voting rights [41]. Some patient and public representatives felt overwhelmed with the use of medical jargon and the number of complex topics discussed in the consortium meetings. We responded to their request for better preparation for the meetings by, for example, circulating the agenda and relevant documents to them beforehand. The feedback given by PPI participants on the project suggests that not all of them felt they had taken a significant part in the process; this notwithstanding, PPI participants appreciated the transparency of the research process and the valuing of their experience and perspectives, and spoke positively of the experience of meeting new and interesting people they would not have had the opportunity to meet otherwise.

### What this study adds

This study has made available a set of best practice recommendations on the use of sedative drugs in specialist inpatient and home palliative care settings, with transparent reporting on their development and their approval by experts.

## Conclusions

The best practice recommendations created in this study provide palliative care professionals with a legally and ethically sound basis for the use of sedative drugs and intentional sedation at the end of life and may help support professionals in making challenging decisions. We believe that future work should focus on implementing and testing the feasibility of a complex intervention, designed on the basis of these recommendations, in specialist end-of-life care settings. It should further seek to assess the effectiveness of this intervention and enable greater participation for representatives of patients and the wider public.

### Supplementary Information

**Additional file 1.**

## Data Availability

Our data protection information to participants guaranteed their anonymity in any presentation or dissemination of the study’s findings. As the participants’ responses contained detailed information on their individual experiences and views on the practice of sedative use in palliative care, it is not possible to anonymise this data completely. The raw data are therefore unavailable. Please contact the corresponding author with specific enquiries; she may be able, within reason, to extract data relating to specific research questions.
