# Supplementary material for: Expert-approved best practice recommendations on the use of sedative drugs and intentional sedation in specialist palliative care (SedPall)
Source: BMC Palliat Care. 2023 Sep 4;22:126. doi: 10.1186/s12904-023-01243-z (PMC10476406; doi:10.1186/s12904-023-01243-z)
Supplement: Supplementary file 1 — Additional file 1. [file 12904_2023_1243_MOESM1_ESM.pdf]

Suppl.: Single-round Delphi in the form of an online survey (English translation and German original)

Instruction

Dear expert,
thank you very much for participating in our Delphi study.
In this single-round Delphi, we will present recommendations sorted according to topics with the request to agree or disagree with it. In the event of disagreement or if you have any other comments on the recommendations, we would be grateful if you could give a brief comment. For each recommendation, you will find the applicable setting: SIPC = Specialist Inpatient Palliative Care, SPHC = Specialist Palliative Home Care.
By clicking on the "back"-button, you can return to questions that have already been answered at any time and adjust your answer. When closing the survey or resuming later, the survey will open on the last edited page.
Your comments and decisions (agreement/disagreement) will be anonymized and prepared for a consensus conference. In the consensus conference, we would like to discuss with you the recommendations that have been approved by less than 75% of all experts in the survey.
We thank you very much for your participation and your efforts.

**Theme**		**Recommendation**	**Setting**	**Agreement/ Disagreement**	**Comment**
Indication	1	Before sedating medication is used, the indication must be defined and documented.	SIPC, SPHC	□ Agree<br>□ Disagree	
	2	Sedating medication can be used to relieve symptoms that patients find distressing, such as anxiety and agitation, without intending to alter consciousness.	SIPC, SPHC	□ Agree<br>□ Disagree	
	3	Sedating medication can be used to relieve insomnia, if experienced as distressing by the patient. In this context, a temporary and reversible change in consciousness is intended (RASS-PAL <0)[1].	SIPC, SPHC	□ Agree<br>□ Disagree	
	4	Sedating medication can be administered to prevent suffering during or upon termination of medical measures.	SIPC, SPHC	□ Agree<br>□ Disagree	
	5	In the case of distressing symptoms which, despite all proportionate measures to relieve them (measures administered on expert level), have not been sufficiently alleviated and remain unbearable for the patient, intentional sedation is indicated.	SIPC, SPHC	□ Agree<br>□ Disagree	
	6	In medical crisis situations, such as acute haemorrhage or acute obstruction of the respiratory tract, in addition to opioid treatment of possible dyspnoea, intentional - if necessary deep - sedation is indicated.	SIPC, SPHC	□ Agree<br>□ Disagree	
	7	Existential suffering is not an indication for deep continuous sedation until death without prior temporary sedation.	SIPC, SPHC	□ Agree<br>□ Disagree	

	8	In general, the maintenance of deep sedation until death is only indicated when it can be assumed - with almost complete certainty - that a reduction in the level of sedation would lead to unbearable suffering again.	SIPC, SPHC	□ Agree<br>□ Disagree
	9	The wish to die is not an indication for the administration of potentially sedating medication and therefore also not for intentional sedation.	SIPC, SPHC	□ Agree<br>□ Disagree
	10	The desire for sedation is not an indication for the use of potentially sedating medication and, no indication for intentional sedation.	SIPC, SPHC	□ Agree<br>□ Disagree
Intent/ Purpose	11	Sedating medication should be administered for the purpose of relieving symptoms, relieving suffering, or preventing imminent suffering during or upon termination of medical measures.	SIPC, SPHC	□ Agree<br>□ Disagree
	12	Before and during intentional sedation, the team ensures that the suffering of the patient remains the central focus and that the sedating medication is not used for the purpose of reducing the burden on the family or the team.	SIPC, SPHC	□ Agree<br>□ Disagree
	13	Intentional sedation must not be administered to hasten death of the patient.	SIPC, SPHC	□ Agree<br>□ Disagree
	14	Intentional sedation which results in a limitation of mobility, may (only) be administered without judicial authorisation if the prevention of leaving the place of residence is not the primary purpose but a side effect of the primary intended relief of suffering.	SIPC, SPHC	□ Agree<br>□ Disagree
Decision-making	15	The decision to use intentional sedation will be made in accordance with the (presumed) will of the patient.	SIPC, SPHC	□ Agree<br>□ Disagree
	16	Before intentional sedation, the patient or their legal representative and the treatment team must determine who is involved in the decision-making process.	SIPC, SPHC	□ Agree<br>□ Disagree
	17	In the case of diseases in which severe respiratory distress and/or a haemorrhage can be expected (e.g. tumours of the head or neck, motor neurone disease, COPD, pulmonary fibrosis), the option of symptom-relieving intentional sedation should be discussed in advance with the patient or their legal representative.<br>This conversation should be documented in the patient's record or health care planning documentation for the last phase of life.	SIPC, SPHC	□ Agree<br>□ Disagree
	18	The assessment of whether symptoms remain refractory and unbearable for the patient, despite all proportionate (expert delivered) measures to relieve symptoms, takes place during a multi-professional case conference. In	SIPC, SPHC	□ Agree<br>□ Disagree

		cases of existential suffering, psychological and pastoral competencies should be included in the case conference.		
	19	In cases of ethical conflict, the decision-making process relating to whether or not intentional sedation is to be administered should be supported by ethics counselling/an ethics case conference. Ethics counselling/ethics case conferences must be transparently documented in the patient's record.	SIPC, SPHC	□ Agree<br>□ Disagree
	20	If intentional sedation is initiated during acute episodes of symptom exacerbation, when multi-professional discussion of the case is not possible, then this must be retrospectively carried out as soon as possible to confirm or revise the course of treatment.	SIPC, SPHC	□ Agree<br>□ Disagree
	21	If the use of a medication results in an unwanted reduction in consciousness, then an adjustment to the medication (dose, substance) to reverse the reduction in consciousness is to be considered or a decision must be made promptly at a case conference as to whether intentional sedation is indicated and corresponds to the (presumed) will of the patient. Only then intentional sedation - using suitable medication - is deemed appropriate.	SIPC, SPHC	□ Agree<br>□ Disagree
	22	The decision-making process for intentional sedation, the parties involved in the decision-making process, and the results of the decisions must be transparently documented in the patient's record.	SIPC, SPHC	□ Agree<br>□ Disagree
Information/Consent	23	Before intentional sedation, the patient or their legal representative will be informed of all relevant indications, intentions, effects, planned duration, adverse effects, risks, potential effects on length of life (both in regard to shortening or prolongation), possible course without sedation, and voluntary nature of consent to the sedation.	SIPC, SPHC	□ Agree<br>□ Disagree
	24	When using medication that is not specifically used for sedation but may cause sedation as a side effect, the patient or their legal representative will be informed of this risk.	SIPC, SPHC	□ Agree<br>□ Disagree
	25	The treatment team must involve the patient's relatives in the process of providing information on the intentional sedation if this is the wish of the patient or their legal representative.	SIPC, SPHC	□ Agree<br>□ Disagree
	26	The patient, and with the patient's consent, their relatives are to be informed that the patient's ability to communicate during the use of sedating medication will be limited, especially in cases of intentional sedation. If the patient no longer possesses the capacity to consent, the legal	SIPC, SPHC	□ Agree<br>□ Disagree

		representative of the patient should receive the necessary information.		
	27	To ensure the patient’s right to self-determination, after providing the relevant information and a suitable time frame, the patient will be asked to consent to administration of intentional sedation (informed consent).<br>If the patient no longer possesses the capacity to consent, the legal representative of the patient should be asked to provide the necessary consent.	SIPC, SPHC	□ Agree<br>□ Disagree
	28	Before the administration of intentional sedation, decisions to be made during the period of (potential) incapacity to consent should be discussed with the patient (if the patient is unable to consent, then with the patient’s legal representative). The discussion covers aspects such as rituals, nursing measures, duration of sedation, targeted level of sedation, possible attempts to awaken the patient (including the possible foregoing of the same), the management of other medications, and (artificial) hydration and nutrition.	SIPC, SPHC	□ Agree<br>□ Disagree
	29	If intentional sedation is initiated during acute episodes of symptom exacerbation, and it is not possible to provide the necessary information, this should be provided as soon as possible, if necessary, by retrospectively informing the patient's legal representative.	SIPC, SPHC	□ Agree<br>□ Disagree
	30	The information process and the type of information provided are to be transparently documented in the patient's record.	SIPC, SPHC	□ Agree<br>□ Disagree
Medication and types of sedation	31	When using sedating medication, the substance selection is based on the indication, intention, effect, and duration of the treatment and possible adverse effects.	SIPC, SPHC	□ Agree<br>□ Disagree
	32	Intentional sedation uses the lowest possible dose of the medication to achieve the level of sedation necessary to relieve the patient’s suffering. Therefore, the dose should always ensure that the patient's suffering is reduced to a level tolerable for the patient and that the sedation level is no deeper than necessary.	SIPC, SPHC	□ Agree<br>□ Disagree
	33	Generally, on initiation a medication dose is chosen to achieve light to moderate sedation (RASS-PAL -1 to -2)[1]. Subsequently, the dose is adjusted in accordance with the recommendation in 2).	SIPC, SPHC	□ Agree<br>□ Disagree
	34	In case of acute crisis (e.g. acute respiratory tract obstruction, severe haemorrhage), an initial medication dose to achieve a deep level of sedation (RASS-PAL ≤ -3)[1] can be selected.	SIPC, SPHC	□ Agree<br>□ Disagree
	35	In the event of changes in respiratory activity (bradypnea, hypoventilation)	SIPC, SPHC	□ Agree

		during intentional sedation, it should be critically assessed whether these changes are due to the dying phase or the medication dose. If the medication dose is found to be the cause of the change in respiration, then a dose reduction adapted to the relief of suffering should be considered. If the reduction in respiratory activity is due to the dying phase, then no dose reduction is necessary.		□ Disagree
	36	Intentional sedation should initially be administered as temporary sedation for a pre-determined period of time (up to a maximum of 24 hours). Then, the dose of the sedating medication is reduced or the sedation is discontinued and the situation will be re-evaluated.	SIPC, SPHC	□ Agree<br>□ Disagree
	37	Intentional sedation in case of existential suffering is initially administered as temporary sedation for a predefined time period (up to a maximum of 24 hours).	SIPC, SPHC	□ Agree<br>□ Disagree
	38	Benzodiazepines, e.g. midazolam, are suitable for intentional sedation . Generally, these medications are the first choice, especially for patients requiring a reduction in anxiety levels and/or anti-epileptic effects. In the case of delirium, they should only be administered in combination with antipsychotic medication.	SIPC, SPHC	□ Agree<br>□ Disagree
	39	Antipsychotics with sedating (secondary) effects, e.g. levomepromazine, are a suitable second choice medication for intentional sedation. They can be administered in combination with benzodiazepines in cases in which benzodiazepines alone are inadequate to achieve sufficient relief of suffering.	SIPC, SPHC	□ Agree<br>□ Disagree
	40	Propofol is suitable for intentional sedation in cases in which other types of medication have not resulted in sufficient relief of suffering.	SIPC	□ Agree<br>□ Disagree
	41	Propofol is not suitable for intentional sedation in the home care setting.	SPHC	□ Agree<br>□ Disagree
	42	Opioids are not suitable for use in intentional sedation. Increasing the dose of an existing opioid therapy is also not a suitable means of intentional sedation. During intentional sedation, opioid treatment to reduced pain levels and/or treat dyspnoea is continued and the dose is adjusted as needed to ensure relief of pain and/or dyspnoea.	SIPC, SPHC	□ Agree<br>□ Disagree
Monitoring	43	During sedation, the situation is re-evaluated by the person administering treatment and the dose adjusted to ensure the suffering is relieved to an acceptable level and that the level of sedation is no more than that required	SIPC, SPHC	□ Agree<br>□ Disagree

		to relief the suffering.		
	44	The criteria for regular re-evaluation of the overall situation are intensity of suffering (most important criterion), level of sedation, and adverse effects.	SIPC, SPHC	□ Agree<br>□ Disagree
	45	The person administering intentional sedation is expected to use the patient’s relatives as an important supplementary source of information during regular re-evaluation.	SIPC, SPHC	□ Agree<br>□ Disagree
	46	During intentional sedation, depending on the illness situation and the treatment goals, selected vital signs (e.g. respiratory rate, oxygen saturation, heart rate, and blood pressure) could additionally be monitored to ensure a stable clinical status of the patient within the framework of the agreed objectives and limits of treatment. Threshold values and corresponding consequences and reactions must be defined for monitored vital signs.	SIPC, SPHC	□ Agree<br>□ Disagree
	47	During deep sedation outside of the dying phase, appropriate (vital) signs and parameters should be monitored to ensure that shortening of life is avoided as far as possible.	SIPC, SPHC	□ Agree<br>□ Disagree
	48	The frequency of re-evaluation should be determined (and adjusted, as necessary) by the physician responsible for the intentional sedation, taking into consideration the planned type of sedation and the pharmacokinetic properties of the sedating medication. Differences between titration phase and maintenance phases have to be considered.	SIPC, SPHC	□ Agree<br>□ Disagree
	49	As far as possible, the intensity of suffering should be assessed by directly asking the patient or their relatives, as well as by clinical observation (e.g. facial expression, sounds like groaning and screaming, body language, movements, agitation, tachycardia, and sweating).	SIPC, SPHC	□ Agree<br>□ Disagree
	50	The depth intentional sedation is assessed based on reactions to being addressed and light, non-painful touching e.g. using RASS-PAL[1].	SIPC, SPHC	□ Agree<br>□ Disagree
	51	The results of the re-evaluation of intentional sedation and the resulting consequences must be transparently documented in the patient’s record.	SIPC, SPHC	□ Agree<br>□ Disagree
Management of fluids and nutrition	52	The decision to administer artificial hydration and/or nutrition must be made before or during sedation if the patient will no longer be able to eat and drink sufficiently on their own.	SIPC, SPHC	□ Agree<br>□ Disagree
	53	The decision to determine whether the artificial administration of fluids and/or nutrition is indicated must be made separately from the decision on intentional sedation.	SIPC, SPHC	□ Agree<br>□ Disagree

	54	In the case of intentional sedation, any decision on artificial hydration and/or nutrition is made with the patient or the patient´s legal representative or based on the presumed will of the patient and taking into consideration possible advantages and burdens as a result of these measures with regard to the treatment goals (relief of suffering).	SIPC, SPHC	□ Agree<br>□ Disagree
	55	The decision relating to artificial hydration and/or nutrition during intentional sedation should be transparently documented in the patient's record.	SIPC, SPHC	□ Agree<br>□ Disagree
Continuing other measures	56	During intentional sedation, the patient will continue to be treated in the same dignified manner as before sedation. This includes addressing the patient (also in phases during which the patient is not conscious), announcing in advance actions that involve touching the patient, and adapting the surroundings to the given situation and, if necessary, in accordance with the previously discussed wishes of the patient.	SIPC, SPHC	□ Agree<br>□ Disagree
	57	All nursing and medical measures are to be regularly evaluated and orientated towards the well-being of the patient. The measures should be adjusted to the changing conditions during intentional sedation and in accordance with the stated or presumed will of the patient.	SIPC, SPHC	□ Agree<br>□ Disagree
	58	Measures to ensure symptom relief and patient well-being that were implemented before the intentional sedation are normally continued, regularly re-evaluated, and adjusted if necessary.	SIPC, SPHC	□ Agree<br>□ Disagree
Support for relatives	59	With the consent of the patient, the relatives should be included from the beginning in the decision-making process related to intentional sedation.	SIPC, SPHC	□ Agree<br>□ Disagree
	60	With the consent of the patient, relatives will be regularly informed of the patient’s current clinical situation and the expected course throughout the intentional sedation.	SIPC, SPHC	□ Agree<br>□ Disagree
	61	The team offers support to the relatives regarding their emotional or spiritual needs resulting from the intentional sedation.	SIPC, SPHC	□ Agree<br>□ Disagree
	62	The relatives are advised and, if necessary, instructed on how to support the patient during the intentional sedation and remain close to them, e.g. by talking, touching, creating a comforting atmosphere for the patient (e.g. favourite music, smells, singing well-known songs, reading aloud) and, if desired - are involved in the nursing care (e.g. mouth care).	SIPC, SPHC	□ Agree<br>□ Disagree
	63	Before deep sedation, which is expected to continue until death, or sedation which may become deep continuous sedation, the patient and their relatives	SIPC, SPHC	□ Agree<br>□ Disagree

		should be given the opportunity to say goodbye to one another if the situation allows it.		
	64	After the death of the patient, the relatives will be given the opportunity to talk to members of the treatment team to discuss any remaining doubts concerning the intentional sedation.	SIPC, SPHC	□ Agree<br>□ Disagree
Team support	65	All team members must fully understand the indications and treatment objectives of intentional sedation. The necessary discussions can take place at team meetings or during case conferences.	SIPC, SPHC	□ Agree<br>□ Disagree
	66	The discussion of stressful situations relating to intentional sedation, e.g. a retrospective case review or conference, is recommended. The aim of these meetings is to discuss the factual and emotional challenges, help the team process stress, and continuously improve the care provided.	SIPC, SPHC	□ Agree<br>□ Disagree
Overall comment:				
Demographic data	How old are you?			
	Please choose your professional background.		□ physician<br>□ nurse<br>□ psychologist<br>□ physiotherapist<br>□ social worker<br>□ others (please name)	
	Please choose setting. (multiple answers possible)		□ Specialist Inpatient Palliative Care<br>□ Specialist Palliative Home Care	

Legend: SIPC = Specialist Inpatient Palliative Care, SPHC = Specialist Palliative Home Care; Please find the original German version of the single-round Delphi online survey below. English translation by the authors. 1 Bush et al. 2014

Fragebogen

1 Standardseite

Liebe Expertinnen und Experten,

vielen herzlichen Dank, dass Sie an der Umfrage und der Konsentierung der Handlungsempfehlung "Einsatz sedierender Medikamente in der Spezialisierten Palliativversorgung" mitwirken.

Im Nachfolgenden wird Ihnen unter Angabe des jeweiligen Themas immer eine einzelne Handlungsempfehlung präsentiert - mit der Bitte, dieser zuzustimmen oder diese abzulehnen. Bei Ablehnung oder sonstigen Hinweisen zur Handlungsempfehlung sind wir Ihnen über einen kurzen Kommentar dankbar. Für jede Handlungsempfehlung finden Sie auch einen entsprechend festgelegten Geltungsbereich (SSPV = Stationäre Spezialisierte Palliativversorgung; SAPV = Spezialisierte Ambulante Palliativversorgung).

Über den "Zurück-Button" können Sie jederzeit zu bereits beantworteten Fragen zurückkehren und Ihre dortigen Antworten anpassen. Hierzu müssen Sie allerdings bei der aktuellen Frage eine Antwortmöglichkeit auswählen, da ansonsten die Filternutzung blockiert. Beim Schließen der Umfrage oder dem Fortsetzen zu einem späteren Zeitpunkt, öffent sich die Umfrage auf der zuletzt bearbeiteten Seite.

Ihre Anmerkungen und Einschätzungen werden anschließend - selbstverständlich anonymisiert - für die Konsensuskonferenz am 10.02.2021 aufbereitet. In der Konsensuskonferenz möchten wir dann gemeinsam mit Ihnen noch ausschließlich die Handlungsempfehlungen besprechen, die in der vorliegenden Umfrage von weniger als 75 Prozent aller am Konsensusprozess teilnehmenden ExpertInnen Zustimmung erhalten haben. Wir bitten möglichst jede/n Einzelne/n von Ihnen um Ihre Teilnahme, damit wir die Zeit auf der Konsensuskonferenz optimal nutzen können.

Wir danken Ihnen sehr für Ihre Teilnahme und Ihr Engagement!

Herzliche Grüße im Namen des Verbundes
Prof. Dr. Christoph Ostgathe

P.S.: Da es teilweise enge inhaltliche und strukturelle Wechselbeziehungen zwischen den einzelnen Handlungsempfehlungen gibt, könnte es sein, dass auch im Nachgang noch einmal angleichende Veränderungen an die schon konsentierten Handlungsempfehlungen vorgenommen werden müssen.

2 Standardseite - Indikation

Es folgen Handlungsempfehlungen zum Thema: Indikation.

3 Standardseite

Thema: Indikation, Nr. 1

Vor dem Einsatz sedierender Medikamente muss die Indikation festgelegt und dokumentiert werden.

Geltungsbereich: SSPV, SAPV.

Bitte geben Sie an, ob Sie der Handlungsempfehlung zustimmen oder diese ablehnen.

- ◯ Zustimmung
- ◯ Ablehnung

Kommentarfeld

4 Standardseite

Thema: Indikation, Nr. 2

Sedierende Medikamente können zur Linderung der von der Patientin/dem Patienten als belastend empfundenen Symptome wie Angst und Unruhe dienen, ohne dass auf eine Änderung des Bewusstseins abgezielt wird.

Geltungsbereich: SSPV, SAPV.

Bitte geben Sie an, ob Sie der Handlungsempfehlung zustimmen oder diese ablehnen.

- ◯ Zustimmung
- ◯ Ablehnung

Kommentarfeld

5 Standardseite

Thema: Indikation, Nr. 3

Sedierende Medikamente können der Linderung einer von der Patientin/dem Patienten als belastend empfundenen Schlaflosigkeit dienen; hierbei wird eine reversible, zeitweise Änderung des Bewusstseins beabsichtigt (RASS-PAL ab < 0).

Geltungsbereich: SSPV, SAPV.

Bitte geben Sie an, ob Sie der Handlungsempfehlung zustimmen oder diese ablehnen.

○ Zustimmung

○ Ablehnung

Kommentarfeld

6 Standardseite

Thema: Indikation, Nr. 4

Sedierende Medikamente können zur Vorbeugung von Leiden bei der Durchführung oder Beendigung einer medizinischen Maßnahme eingesetzt werden.

Geltungsbereich: SSPV, SAPV.

Bitte geben Sie an, ob Sie der Handlungsempfehlung zustimmen oder diese ablehnen.

○ Zustimmung

○ Ablehnung

Kommentarfeld

7 Standardseite

Thema: Indikation, Nr. 5

Bei belastenden Symptomen, die trotz aller verhältnismäßigen Maßnahmen zur Symptomlinderung (Maßnahmen auf Expertinnen-/Expertenniveau) nicht ausreichend gelindert wurden und für die Patientin/den Patienten unerträglich sind, ist eine gezielte Sedierung indiziert.

Geltungsbereich: SSPV, SAPV.

Bitte geben Sie an, ob Sie der Handlungsempfehlung zustimmen oder diese ablehnen.

- ○ Zustimmung
- ○ Ablehnung

Kommentarfeld

8 Standardseite

Thema: Indikation, Nr. 6

In krisenhaften Situationen wie bei einer akuten schweren Blutung oder einer akuten Verlegung der Atemwege ist zusätzlich zur Therapie etwaiger Dyspnoe mittels Opioiden eine gezielte - gegebenenfalls tiefe - Sedierung indiziert.

Geltungsbereich: SSPV, SAPV.

Bitte geben Sie an, ob Sie der Handlungsempfehlung zustimmen oder diese ablehnen.

- ◯ Zustimmung
- ◯ Ablehnung

Kommentarfeld

9 Standardseite

Thema: Indikation, Nr. 7

Existenzielles Leiden ist keine Indikation für eine tiefe kontinuierliche Sedierung bis zum Tod, ohne dass zuvor eine vorübergehende Sedierung durchgeführt wurde.

Geltungsbereich: SSPV, SAPV.

Bitte geben Sie an, ob Sie der Handlungsempfehlung zustimmen oder diese ablehnen.

- ◯ Zustimmung
- ◯ Ablehnung

Kommentarfeld

10 Standardseite

Thema: Indikation, Nr. 8

Allgemein ist das Aufrechterhalten einer tiefen Sedierung bis zum Tod nur dann indiziert, wenn mit an Sicherheit grenzender Wahrscheinlichkeit davon auszugehen ist, dass eine Reduktion der Sedierungstiefe erneut zu unerträglichem Leiden führen würde.

Geltungsbereich: SSPV, SAPV.

Bitte geben Sie an, ob Sie der Handlungsempfehlung zustimmen oder diese ablehnen.

○ Zustimmung

○ Ablehnung

Kommentarfeld

11 Standardseite

Thema: Indikation, Nr. 9

Ein Wunsch zu Sterben ist keine Indikation für den Einsatz potenziell sedierender Medikamente und somit auch nicht für eine gezielte Sedierung.

Geltungsbereich: SSPV, SAPV.

Bitte geben Sie an, ob Sie der Handlungsempfehlung zustimmen oder diese ablehnen.

○ Zustimmung

○ Ablehnung

Kommentarfeld

12 Standardseite

Thema: Indikation, Nr. 10

Ein Wunsch nach Sedierung ist keine Indikation für den Einsatz potenziell sedierender Medikamente und somit auch nicht für eine gezielte Sedierung.

Geltungsbereich: SSPV, SAPV.

Bitte geben Sie an, ob Sie der Handlungsempfehlung zustimmen oder diese ablehnen.

- ○ Zustimmung
- ○ Ablehnung

Kommentarfeld

13 Standardseite - Intention/Zweck

Es folgen Handlungsempfehlungen zum Thema: Intention/Zweck.

14 Standardseite

Thema: Intention/Zweck, Nr. 1

Sedierende Medikamente sollen zum Zweck der Symptomlinderung, der Leidenslinderung oder der Vorbeugung unmittelbar bevorstehenden Leidens bei der Durchführung oder Beendigung einer medizinischen Maßnahme eingesetzt werden.

Geltungsbereich: SSPV, SAPV.

Bitte geben Sie an, ob Sie der Handlungsempfehlung zustimmen oder diese ablehnen.

- ○ Zustimmung
- ○ Ablehnung

Kommentarfeld

15 Standardseite

Thema: Intention/Zweck, Nr. 2

Das Team trägt vor und während der gezielten Sedierung dafür Sorge, dass das Leid der Patientin/des Patienten im Fokus steht und der Einsatz sedierender Medikamente nicht zur Linderung von Belastungen der Zugehörigen oder des Teams dient.

Geltungsbereich: SSPV, SAPV.

Bitte geben Sie an, ob Sie der Handlungsempfehlung zustimmen oder diese ablehnen.

- ○ Zustimmung
- ○ Ablehnung

Kommentarfeld

16 Standardseite

Thema: Intention/Zweck, Nr. 3

Gezielte Sedierung darf nicht zur Beschleunigung des Todeseintritts eingesetzt werden.

Geltungsbereich: SSPV, SAPV.

Bitte geben Sie an, ob Sie der Handlungsempfehlung zustimmen oder diese ablehnen.

- ◯ Zustimmung
- ◯ Ablehnung

Kommentarfeld

17 Standardseite

Thema: Intention/Zweck, Nr. 4

Gezielte Sedierung, die eine Beschränkung der Fortbewegungsfreiheit zur Folge hat, darf ohne richterliche Genehmigung (nur) erfolgen, wenn die Hinderung am Verlassen des Aufenthaltsortes nicht primärer Zweck, sondern Nebenwirkung einer vorrangig bezweckten Leidenslinderung ist.

Geltungsbereich: SSPV, SAPV.

Bitte geben Sie an, ob Sie der Handlungsempfehlung zustimmen oder diese ablehnen.

- ◯ Zustimmung
- ◯ Ablehnung

Kommentarfeld

18 Standardseite - Entscheidungsprozess

Es folgen Handlungsempfehlungen zum Thema: Entscheidungsprozess.

19 Standardseite

Thema: Entscheidungsprozess, Nr. 1

Die Entscheidung, ob eine gezielte Sedierung eingesetzt wird, erfolgt unter Beachtung des (mutmaßlichen) Willens der Patientin/des Patienten.

Geltungsbereich: SSPV.

Bitte geben Sie an, ob Sie der Handlungsempfehlung zustimmen oder diese ablehnen.

- ○ Zustimmung
- ○ Ablehnung

Kommentarfeld

20 Standardseite

Thema: Entscheidungsprozess, Nr. 2

Für eine gezielte Sedierung ist von der Patientin/dem Patienten bzw. der rechtlichen Vertreterin/des rechtlichen Vertreters und dem Behandlungsteam festzulegen, wer in den Entscheidungsprozess involviert wird.

Geltungsbereich: SSPV, SAPV.

Bitte geben Sie an, ob Sie der Handlungsempfehlung zustimmen oder diese ablehnen.

- ◯ Zustimmung
- ◯ Ablehnung

Kommentarfeld

21 Standardseite

Thema: Entscheidungsprozess, Nr. 3

Bei Krankheiten, bei denen mit starker Atemnot und/oder akuter starker Blutung gerechnet werden muss (z.B. Kopf-Hals-Tumore, amyotrophe Lateralsklerose, COPD, Lungenfibrose) ist ggf. die Möglichkeit einer gezielten Sedierung zur Symptomlinderung vorab mit der Patientin/dem Patienten bzw. deren rechtlichen Stellvertreterin/ dessen rechtlichem Stellvertreter zu besprechen. Dieses Gespräch ist in der Patientinnen-/Patientenakte, bspw. in den Dokumenten zur Gesundheitlichen Versorgungplanung für die letzte Lebensphase festzuhalten.

Geltungsbereich: SSPV, SAPV.

Bitte geben Sie an, ob Sie der Handlungsempfehlung zustimmen oder diese ablehnen.

- ◯ Zustimmung
- ◯ Ablehnung

Kommentarfeld

22 Standardseite

Thema: Entscheidungsprozess, Nr. 4

Die Einschätzung, ob Symptome trotz aller verhältnismäßigen Maßnahmen zur Symptomlinderung (Maßnahmen auf Expertinnen-/Expertenniveau) therapierefraktär und für die Patientin/den Patienten unerträglich geblieben sind, erfolgt im Rahmen einer multiprofessionellen Fallbesprechung. Bei existenziellem Leiden ist psychologische und seelsorgerische Kompetenz für die Fallbesprechung einzubinden.

Geltungsbereich: SAPV, SSPV.

Bitte geben Sie an, ob Sie der Handlungsempfehlung zustimmen oder diese ablehnen.

- ○ Zustimmung
- ○ Ablehnung

Kommentarfeld

23 Standardseite

Thema: Entscheidungsprozess, Nr. 5

Bei ethischen Konflikten sollte die Entscheidungsfindung, ob eine gezielte Sedierung durchgeführt wird, durch eine Ethikberatung/ein ethisches Fallgespräch unterstützt werden. Die Ethikberatung/das ethische Fallgespräch werden

nachvollziehbar in der Patientinnen-/Patientenakte dokumentiert.

Geltungsbereich: SSPV, SAPV.

Bitte geben Sie an, ob Sie der Handlungsempfehlung zustimmen oder diese ablehnen.

○ Zustimmung

○ Ablehnung

Kommentarfeld

24 Standardseite

Thema: Entscheidungsprozess, Nr. 6

Wird eine gezielte Sedierung in einer akut krisenhaften Symptomexazerbation initiiert, in der eine Absprache im Rahmen einer multiprofessionellen Fallbesprechung nicht möglich ist, ist diese zeitnah nachzuholen und die Entscheidung zu bestätigen oder zu revidieren.

Geltungsbereich: SSPV, SAPV.

Bitte geben Sie an, ob Sie der Handlungsempfehlung zustimmen oder diese ablehnen.

○ Zustimmung

○ Ablehnung

Kommentarfeld

25 Standardseite

Thema: Entscheidungsprozess, Nr. 7

Tritt durch Einsatz eines Medikamentes unbeabsichtigt eine Bewusstseinsreduktion ein, ist eine Anpassung der Medikation (Dosis, Substanz) zur Rücknahme der Bewusstseinsreduktion zu überprüfen oder im Rahmen einer multiprofessionellen Fallbesprechung zeitnah zu entscheiden, ob eine gezielte Sedierung indiziert ist und dem (mutmaßlichen) Willen der Patientin/des Patienten entspricht. Nur dann ist eine gezielte Sedierung, unter Anwendung hierfür geeigneter Medikation, angemessen.

Geltungsbereich: SSPV, SAPV.

Bitte geben Sie an, ob Sie der Handlungsempfehlung zustimmen oder diese ablehnen.

- ○ Zustimmung
- ○ Ablehnung

Kommentarfeld

26 Standardseite

Thema: Entscheidungsprozess, Nr. 8

Der Prozess der Entscheidung für die gezielte Sedierung, die Beteiligten im Entscheidungsprozess und das Ergebnis der Entscheidung sind nachvollziehbar in der Patientinnen-/Patientenakte zu dokumentieren.

Geltungsbereich: SSPV, SAPV.

Bitte geben Sie an, ob Sie der Handlungsempfehlung zustimmen oder diese ablehnen.

- ◯ Zustimmung
- ◯ Ablehnung

Kommentarfeld

27 Standardseite - Aufklärung/Einwilligung

Es folgen Handlungsempfehlungen zum Thema: Aufklärung/Einwilligung.

28 Standardseite

Thema: Aufklärung/Einwilligung, Nr. 1

Die Patientin/der Patient oder deren/dessen rechtliche Stellvertreterin/rechtlicher Stellvertreter wird vor der gezielten Sedierung über Indikation, Intention, Wirkung, geplante Dauer, Nebenwirkung, Risiken, potentielle Auswirkung auf die Lebenszeit (sowohl hinsichtlich einer Verkürzung als auch einer Verlängerung), möglichen Verlauf ohne Sedierung und Freiwilligkeit der Inanspruchnahme aufgeklärt.

Geltungsbereich: SSPV, SAPV.

Bitte geben Sie an, ob Sie der Handlungsempfehlung zustimmen oder diese ablehnen.

- ◯ Zustimmung
- ◯ Ablehnung

Kommentarfeld

29 Standardseite

Thema: Aufklärung/Einwilligung, Nr. 2

Bei der Anwendung von Medikamenten, die nicht gezielt zur Sedierung eingesetzt werden, eine solche jedoch als Nebenwirkung bedingen können, ist die Patientin/der Patient oder deren/dessen rechtliche Stellvertreterin/rechtlicher Stellvertreter über dieses Risiko aufzuklären.

Geltungsbereich: SSPV, SAPV.

Bitte geben Sie an, ob Sie der Handlungsempfehlung zustimmen oder diese ablehnen.

- ○ Zustimmung
- ○ Ablehnung

Kommentarfeld

30 Standardseite

Thema: Aufklärung/Einwilligung, Nr. 3

In den Prozess der Aufklärung über gezielte Sedierung sind vom Behandlungsteam, wenn es dem Wunsch der Patientin/des Patienten bzw. deren/dessen rechtlicher Stellvertreterin/rechtlichem Stellvertreter entspricht, die Zugehörigen mit einzubeziehen.

Geltungsbereich: SSPV, SAPV.

Bitte geben Sie an, ob Sie der Handlungsempfehlung zustimmen oder diese ablehnen.

- ◯ Zustimmung
- ◯ Ablehnung

Kommentarfeld

31 Standardseite

Thema: Aufklärung/Einwilligung, Nr. 4

Die Patientin/der Patient und - bei Einverständnis der Patientin/des Patienten - die Zugehörigen sind darüber aufzuklären, dass die Kommunikationsfähigkeit während des Einsatzes sedierender Medikamente eingeschränkt sein kann, insbesondere dann, wenn eine gezielte Sedierung durchgeführt werden soll. Bei fehlender Einwilligungsfähigkeit der Patientin/des Patienten ist die rechtliche Stellvertreterin/der rechtliche Stellvertreter entsprechend aufzuklären.

Geltungsbereich: SSPV, SAPV.

Bitte geben Sie an, ob Sie der Handlungsempfehlung zustimmen oder diese ablehnen.

- ◯ Zustimmung
- ◯ Ablehnung

Kommentarfeld

32 Standardseite

Thema: Aufklärung/Einwilligung, Nr. 5

Zur Wahrung des Selbstbestimmungsrechtes der Patientin/des Patienten erfolgt nach Aufklärung und einem angemessenen Zeitfenster die Einholung des Einverständnisses zum Einsatz gezielter Sedierung (informiertes Einverständnis). Bei fehlender Einwilligungsfähigkeit ist die Einwilligung der rechtlichen Stellvertreterin/des rechtlichen Stellvertreters entsprechend einzuholen.

Geltungsbereich: SSPV, SAPV.

Bitte geben Sie an, ob Sie der Handlungsempfehlung zustimmen oder diese ablehnen.

- ○ Zustimmung
- ○ Ablehnung

Kommentarfeld

33 Standardseite

Thema: Aufklärung/Einwilligung, Nr. 6

Vor einer gezielten Sedierung sind mit der Patientin/dem Patienten (bei fehlender Einwilligungsfähigkeit mit der rechtlichen Stellvertreterin/dem rechtlichen Stellvertreter) Entscheidungen für die Zeit der (eventuellen) Nicht-Einwilligungsfähigkeit zu besprechen. Die Absprachen umfassen Aspekte wie Rituale, pflegerische Maßnahmen, Dauer der Gabe sedierender Medikamente, angestrebte Tiefe der Sedierung, eventuelle Aufwachversuche (inkl. im Verlauf möglicher Verzicht auf geplante Aufwachversuche), Umgang mit sonstiger Medikation und Umgang mit (künstlicher) Nahrung und Flüssigkeit.

Geltungsbereich: SSPV, SAPV.

Bitte geben Sie an, ob Sie der Handlungsempfehlung zustimmen oder diese ablehnen.

○ Zustimmung

○ Ablehnung

Kommentarfeld

34 Standardseite

Thema: Aufklärung/Einwilligung, Nr. 7

Wird eine gezielte Sedierung in einer akut krisenhaften Symptomexazerbation initiiert, in der eine Aufklärung nicht möglich ist, ist diese zeitnah, gegebenenfalls durch Hinzuziehen der rechtlichen Stellvertreterin/des rechtlichen Stellvertreters, nachzuholen.

Geltungsbereich: SSPV, SAPV.

Bitte geben Sie an, ob Sie der Handlungsempfehlung zustimmen oder diese ablehnen.

○ Zustimmung

○ Ablehnung

Kommentarfeld

35 Standardseite

Thema: Aufklärung/Einwilligung, Nr. 8

Prozess und Inhalte der Aufklärung sowie der Einwilligung sind nachvollziehbar in der Patientinnen-/Patientenakte zu dokumentieren.

Geltungsbereich: SSPV, SAPV.

Bitte geben Sie an, ob Sie der Handlungsempfehlung zustimmen oder diese ablehnen.

- ○ Zustimmung
- ○ Ablehnung

Kommentarfeld

36 Standardseite - Medikation und Formen der Sed

Es folgen Handlungsempfehlungen zum Thema: Medikation und Formen der Sedierung.

37 Standardseite

Thema: Medikation und Formen der Sedierung, Nr. 1

Beim Einsatz sedierender Medikamente erfolgt die Auswahl der Substanz nach Indikation, Intention, Wirkung, Wirkdauer und möglichen unerwünschten Wirkungen.

Geltungsbereich: SSPV, SAPV.

Bitte geben Sie an, ob Sie der Handlungsempfehlung zustimmen oder diese ablehnen.

- ○ Zustimmung
- ○ Ablehnung

Kommentarfeld

38 Standardseite

Thema: Medikation und Formen der Sedierung, Nr. 2

Für gezielte Sedierung wird die niedrigstmögliche Dosis des Medikaments bzw. die entsprechende Sedierungstiefe gewählt, die zur Linderung des Leidens notwendig ist. Die Dosis sollte also zu jedem Zeitpunkt so sein, dass das Leiden so weit gelindert ist, dass es für die Patientin/den Patienten erträglich und die Sedierung nicht tiefer als notwendig ist.

Geltungsbereich: SSPV, SAPV.

Bitte geben Sie an, ob Sie der Handlungsempfehlung zustimmen oder diese ablehnen.

- ○ Zustimmung
- ○ Ablehnung

Kommentarfeld

39 Standardseite

Thema: Medikation und Formen der Sedierung, Nr. 3

Im Allgemeinen wird zu Beginn eine Medikamentendosis gewählt, mit der eine leichte bis mäßige Sedierung (RASS-PAL -1 bis -2) erreicht werden soll. Anschließend wird die Dosis unter Berücksichtigung der Empfehlung 2) [vorherige Seite] angepasst.

Geltungsbereich: SSPV, SAPV.

Bitte geben Sie an, ob Sie der Handlungsempfehlung zustimmen oder diese ablehnen.

- ○ Zustimmung
- ○ Ablehnung

Kommentarfeld

40 Standardseite

Thema: Medikation und Formen der Sedierung, Nr. 4

In akuten Krisensituationen (z.B. akute Atemwegsverlegung, starke Blutung) kann von vorne herein eine Medikamentendosis gewählt werden, mit der eine tiefe Sedierung (RASS-PAL ≤ -3) erreicht werden soll.

Geltungsbereich: SSPV, SAPV.

Bitte geben Sie an, ob Sie der Handlungsempfehlung zustimmen oder diese ablehnen.

- ○ Zustimmung
- ○ Ablehnung

Kommentarfeld

41 Standardseite

Thema: Medikation und Formen der Sedierung, Nr. 5

Bei Veränderung der Atemaktivität (Bradypnoe, Hypoventilation) im Rahmen gezielter Sedierung ist kritisch zu überprüfen, ob die Veränderung Ausdruck der Sterbephase oder durch die Medikamentendosis bedingt ist. Falls die Medikamentendosis Ursache der Veränderung der Atemaktivität ist, ist eine an die Leidenslinderung angepasste Dosisreduktion zu erwägen. Falls das allmähliche Abnehmen der Atemaktivität Ausdruck der Sterbephase ist, ist dies kein Grund für eine Dosisreduktion.

Geltungsbereich: SSPV, SAPV

Bitte geben Sie an, ob Sie der Handlungsempfehlung zustimmen oder diese ablehnen.

- ○ Zustimmung
- ○ Ablehnung

Kommentarfeld

---

42 Standardseite

Thema: Medikation und Formen der Sedierung, Nr. 6

Die gezielte Sedierung ist zunächst als vorübergehende Sedierung für einen vorab festgelegten Zeitraum (bis maximal 24 Stunden) zu erwägen. Im Anschluss wird die Dosis des sedierenden Medikaments reduziert oder die Sedierung zunächst beendet und die Situation reevaluiert.

Geltungsbereich: SSPV, SAPV.

Bitte geben Sie an, ob Sie der Handlungsempfehlung zustimmen oder diese ablehnen.

- ○ Zustimmung

○ Ablehnung

Kommentarfeld

43 Standardseite

Thema: Medikation und Formen der Sedierung, Nr. 7

Gezielte Sedierung bei existentiellem Leiden ist in jedem Fall zunächst als vorübergehende Sedierung für einen vorab festgelegten Zeitraum (bis maximal 24 Stunden) durchzuführen.

Geltungsbereich: SSPV, SAPV.

Bitte geben Sie an, ob Sie der Handlungsempfehlung zustimmen oder diese ablehnen.

○ Zustimmung

○ Ablehnung

Kommentarfeld

44 Standardseite

Thema: Medikation und Formen der Sedierung, Nr. 8

Benzodiazepine, z.B. Midazolam, sind geeignete Medikamente zur gezielten Sedierung. In der Regel sind sie Mittel der ersten Wahl, insbesondere bei Patientinnen/Patienten, bei denen auch Angst gelindert und/oder ein antiepileptischer Effekt erzielt

werden soll. Bei einem Delir gilt dies nur in Kombination mit einem Antipsychotikum.

Geltungsbereich: SSPV, SAPV.

Bitte geben Sie an, ob Sie der Handlungsempfehlung zustimmen oder diese ablehnen.

- ◯ Zustimmung
- ◯ Ablehnung

Kommentarfeld

45 Standardseite

Thema: Medikation und Formen der Sedierung, Nr. 9

Antipsychotika mit sedierender (Neben-)wirkung, z.B. Levomepromazin, sind geeignete Medikamente zweiter Wahl zur gezielten Sedierung. Sie können in Kombination mit Benzodiazepinen gegeben werden, wenn Benzodiazepine allein nicht zu einer ausreichenden Leidenslinderung führen.

Geltungsbereich: SSPV, SAPV.

Bitte geben Sie an, ob Sie der Handlungsempfehlung zustimmen oder diese ablehnen.

- ◯ Zustimmung
- ◯ Ablehnung

Kommentarfeld

46 Standardseite

Thema: Medikation und Formen der Sedierung, Nr. 10

Propofol ist ein geeignetes Medikament zur gezielten Sedierung, wenn andere Medikamente nicht zu einer ausreichenden Leidenslinderung führen.

Geltungsbereich: SSPV.

Bitte geben Sie an, ob Sie der Handlungsempfehlung zustimmen oder diese ablehnen.

- ○ Zustimmung
- ○ Ablehnung

Kommentarfeld

47 Standardseite

Thema: Medikation und Formen der Sedierung, Nr. 11

In ambulanter Versorgung ist Propofol kein geeignetes Medikament zur gezielten Sedierung.

Geltungsbereich: SAPV.

Bitte geben Sie an, ob Sie der Handlungsempfehlung zustimmen oder diese ablehnen.

- ○ Zustimmung
- ○ Ablehnung

Kommentarfeld

48 Standardseite

Thema: Medikation und Formen der Sedierung, Nr. 12

Opioide sind keine geeigneten Medikamente zur gezielten Sedierung. Auch eine Dosiserhöhung einer bereits bestehenden Opioidtherapie ist kein geeignetes Mittel zur gezielten Sedierung. Während einer gezielten Sedierung wird die Therapie mit Opioiden zur Linderung von Schmerzen und/oder Dyspnoe fortgesetzt und die Dosis entsprechend im Verlauf angepasst, soweit dies zur Linderung von Schmerzen und/oder Dyspnoe notwendig ist.

Geltungsbereich: SSPV, SAPV.

Bitte geben Sie an, ob Sie der Handlungsempfehlung zustimmen oder diese ablehnen.

- ○ Zustimmung
- ○ Ablehnung

Kommentarfeld

49 Standardseite - Monitoring/Überwachung

Es folgen Handlungsempfehlungen zum Thema: Monitoring/Überwachung.

50 Standardseite

Thema: Monitoring/Überwachung, Nr. 1

Während einer Sedierung wird die Situation durch die Behandlerinnen/Behandler regelmäßig reevaluiert und die Dosis so angepasst, dass das Leiden auf ein erträgliches Maß gelindert und die Sedierung nicht tiefer ist, als zur Leidenslinderung notwendig.

Geltungsbereich: SSPV, SAPV.

Bitte geben Sie an, ob Sie der Handlungsempfehlung zustimmen oder diese ablehnen.

- ○ Zustimmung
- ○ Ablehnung

Kommentarfeld

51 Standardseite

Thema: Monitoring/Überwachung, Nr. 2

Kriterien der regelmäßigen Reevaluation der Gesamtsituation sind Intensität des Leidens (wichtigstes Kriterium), Sedierungstiefe und unerwünschte Arzneimittelwirkung.

Geltungsbereich: SSPV, SAPV.

Bitte geben Sie an, ob Sie der Handlungsempfehlung zustimmen oder diese ablehnen.

- ○ Zustimmung

◯ Ablehnung

Kommentarfeld

52 Standardseite

Thema: Monitoring/Überwachung, Nr. 3

Die Behandlerinnen/Behandler sind während einer gezielten Sedierung angehalten, im Rahmen der regelmäßigen Reevaluation die Zugehörigen als eine wichtige ergänzende Informationsquelle zu nutzen.

Geltungsbereich: SSPV, SAPV.

Bitte geben Sie an, ob Sie der Handlungsempfehlung zustimmen oder diese ablehnen.

◯ Zustimmung

◯ Ablehnung

Kommentarfeld

53 Standardseite

Thema: Monitoring/Überwachung, Nr. 4

Bei gezielter Sedierung können je nach Krankheitssituation und Behandlungszielen zusätzlich ausgewählte Vitalparameter (z.B. Atemfrequenz, Sauerstoffsättigung, Herzfrequenz, Blutdruck) überwacht werden, um innerhalb der abgesprochenen

Behandlungsziele und -grenzen eine stabile klinische Situation der Patientin/des Patienten zu gewährleisten. Im Fall der Erhebung von Vitalparametern ist festzulegen, ab welchen Grenzwerten der erhobenen Parameter Konsequenzen gezogen werden, und welche dafür in Betracht kommen.

Geltungsbereich: SSPV, SAPV.

Bitte geben Sie an, ob Sie der Handlungsempfehlung zustimmen oder diese ablehnen.

- ○ Zustimmung
- ○ Ablehnung

Kommentarfeld

54 Standardseite

Thema: Monitoring/Überwachung, Nr. 5

Bei tiefer Sedierung außerhalb der Sterbephase sind zur Steuerung der Medikation geeignete (Vital-)Parameter zu erfassen, um die Medikation so zu steuern, dass eine Lebensverkürzung soweit möglich vermieden wird.

Geltungsbereich: SSPV, SAPV.

Bitte geben Sie an, ob Sie der Handlungsempfehlung zustimmen oder diese ablehnen.

- ○ Zustimmung
- ○ Ablehnung

Kommentarfeld

55 Standardseite

Thema: Monitoring/Überwachung, Nr. 6

Die Häufigkeit der Reevaluation ist von der Ärztin/dem Arzt, die für die gezielte Sedierung verantwortlich ist, unter Berücksichtigung der geplanten Sedierungsart und der Pharmakokinetik des verwendeten sedierenden Medikaments festzulegen und ggf. im weiteren Verlauf zu verändern. Den besonderen Aspekten der Einstellungs- und Erhaltungsphasen ist hierbei Rechnung zu tragen.

Geltungsbereich: SSPV, SAPV.

Bitte geben Sie an, ob Sie der Handlungsempfehlung zustimmen oder diese ablehnen.

- ○ Zustimmung
- ○ Ablehnung

Kommentarfeld

56 Standardseite

Thema: Monitoring/Überwachung, Nr. 7

Die Intensität des Leidens wird, soweit möglich, durch Befragen der Patientin/des Patienten und/oder der Zugehörigen, sowie durch klinische Beobachtung eingeschätzt, u.a. unter Berücksichtigung von Mimik, Lautäußerungen wie Stöhnen und Schreien,

Körperhaltung, Bewegungen, motorische Unruhe, Tachykardie und Schwitzen.

Geltungsbereich: SSPV, SAPV.

Bitte geben Sie an, ob Sie der Handlungsempfehlung zustimmen oder diese ablehnen.

- ○ Zustimmung
- ○ Ablehnung

Kommentarfeld

57 Standardseite

Thema: Monitoring/Überwachung, Nr. 8

Die Sedierungstiefe wird bei gezielter Sedierung anhand von Reaktion auf Ansprache und leichte, nicht-schmerzhafte Berührung eingeschätzt, z.B. anhand des RASS-PAL.

Geltungsbereich: SSPV, SAPV.

Bitte geben Sie an, ob Sie der Handlungsempfehlung zustimmen oder diese ablehnen.

- ○ Zustimmung
- ○ Ablehnung

Kommentarfeld

58 Standardseite

Thema: Monitoring/Überwachung, Nr. 9

Bei gezielter Sedierung sind Ergebnisse der Reevaluation und daraus gezogene Konsequenzen nachvollziehbar in der Patientinnen-/Patientenakte zu dokumentieren.

Geltungsbereich: SSPV, SAPV.

Bitte geben Sie an, ob Sie der Handlungsempfehlung zustimmen oder diese ablehnen.

- ○ Zustimmung
- ○ Ablehnung

Kommentarfeld

59 Standardseite - Umgang mit Flüssigkeit/Ernährung

Es folgen Handlungsempfehlungen zum Thema: Umgang mit Flüssigkeit/Ernährung.

60 Standardseite

Thema: Umgang mit Flüssigkeit und Ernährung, Nr. 1

Eine Entscheidung über die künstliche Zufuhr von Flüssigkeit und/oder Ernährung ist vor oder während der gezielten Sedierung notwendig, falls die Patientin/der Patient nicht in der Lage sein wird, selbst ausreichend zu essen und zu trinken.

Geltungsbereich: SSPV, SAPV.

Bitte geben Sie an, ob Sie der Handlungsempfehlung zustimmen oder diese ablehnen.

- ○ Zustimmung
- ○ Ablehnung

Kommentarfeld

61 Standardseite

Thema: Umgang mit Flüssigkeit und Ernährung, Nr. 2

Die Entscheidung, ob künstliche Zufuhr von Flüssigkeit und/oder Ernährung indiziert ist, ist getrennt von der Entscheidung für die gezielte Sedierung zu treffen.

Geltungsbereich: SSPV, SAPV.

Bitte geben Sie an, ob Sie der Handlungsempfehlung zustimmen oder diese ablehnen.

- ○ Zustimmung
- ○ Ablehnung

Kommentarfeld

62 Standardseite

Thema: Umgang mit Flüssigkeit und Ernährung, Nr. 3

Die Entscheidung über die künstliche Zufuhr von Flüssigkeit und/oder Ernährung wird im Falle einer gezielten Sedierung in Absprache mit der Patientin/dem Patienten oder ihrer/seinem rechtlichen Stellvertreterin/rechtlichen Stellvertreter bzw. anhand des mutmaßlichen Willens der Patientin/dem Patienten und in Abwägung der möglichen Vorteile und Belastungen dieser Maßnahmen angesichts des Behandlungsziels (Leidenslinderung) getroffen.

Geltungsbereich: SSPV, SAPV.

Bitte geben Sie an, ob Sie der Handlungsempfehlung zustimmen oder diese ablehnen.

○ Zustimmung

○ Ablehnung

Kommentarfeld

63 Standardseite

Thema: Umgang mit Flüssigkeit und Ernährung, Nr. 4

Die Entscheidung bezüglich der künstlichen Zufuhr von Flüssigkeit und Ernährung während der gezielten Sedierung ist nachvollziehbar in der Patientinnen-/Patientenakte zu dokumentieren.

Geltungsbereich: SSPV, SAPV.

Bitte geben Sie an, ob Sie der Handlungsempfehlung zustimmen oder diese ablehnen.

○ Zustimmung

○ Ablehnung

Kommentarfeld

64 Standardseite - Fortsetzung sonstiger Maßnahmen

Es folgen Handlungsempfehlungen zum Thema: Fortsetzung sonstiger Maßnahmen.

65 Standardseite

Thema: Fortsetzung sonstiger Maßnahmen, Nr. 1

Während der gezielten Sedierung ist derselbe würdige Umgang mit der Patientin/dem Patienten aufrechtzuerhalten wie vor der Sedierung. Dies beinhaltet u.a., die Patientin/den Patienten anzusprechen (dies gilt auch in Phasen, in denen die Patientin/der Patient nicht bei Bewusstsein ist), Handlungen vor Berührung der Patientin/des Patienten anzukündigen und die Umgebung an die gegebene Situation und ggf. vorher besprochene Wünsche der Patientin/des Patienten anzupassen.

Geltungsbereich: SSPV, SAPV.

Bitte geben Sie an, ob Sie der Handlungsempfehlung zustimmen oder diese ablehnen.

- ○ Zustimmung
- ○ Ablehnung

Kommentarfeld

66 Standardseite

Thema: Fortsetzung sonstiger Maßnahmen, Nr. 2

Alle pflegerischen und medizinischen Maßnahmen sind regelmäßig zu überprüfen und am Wohle der Patientin/des Patienten zu orientieren. Sie sind entsprechend des zuvor geäußerten oder mutmaßlichen Patientinnen-/Patientenwillens an die veränderten Bedingungen während der gezielten Sedierung anzupassen.

Geltungsbereich: SSPV, SAPV.

Bitte geben Sie an, ob Sie der Handlungsempfehlung zustimmen oder diese ablehnen.

○ Zustimmung

○ Ablehnung

Kommentarfeld

67 Standardseite

Thema: Fortsetzung sonstiger Maßnahmen, Nr. 3

Maßnahmen zur Symptomlinderung und für das Wohlbefinden, die vor Beginn der gezielten Sedierung durchgeführt wurden, werden in der Regel fortgesetzt, im weiteren Verlauf regelmäßig reevaluiert und ggf. angepasst.

Geltungsbereich: SSPV, SAPV.

Bitte geben Sie an, ob Sie der Handlungsempfehlung zustimmen oder diese ablehnen.

○ Zustimmung

○ Ablehnung

Kommentarfeld

68 Standardseite - Begleitung der Zugehörigen

Es folgen Handlungsempfehlungen zum Thema: Begleitung der Zugehörigen.

69 Standardseite

Thema: Begleitung der Zugehörigen, Nr. 1

Die Zugehörigen werden nach Zustimmung durch die Patientin/den Patienten möglichst von Anfang an in den Entscheidungsprozess und die Aufklärung über die gezielte Sedierung einbezogen.

Geltungsbereich: SSPV, SAPV.

Bitte geben Sie an, ob Sie der Handlungsempfehlung zustimmen oder diese ablehnen.

- ○ Zustimmung
- ○ Ablehnung

Kommentarfeld

70 Standardseite

Thema: Begleitung der Zugehörigen, Nr. 2

Während der gezielten Sedierung werden die Zugehörigen entsprechend des Patientinnen-/Patientenwunsches regelmäßig über die aktuelle klinische Situation und den weiter zu erwartenden Verlauf informiert.

Geltungsbereich: SSPV, SAPV.

Bitte geben Sie an, ob Sie der Handlungsempfehlung zustimmen oder diese ablehnen.

- ◯ Zustimmung
- ◯ Ablehnung

Kommentarfeld

71 Standardseite

Thema: Begleitung der Zugehörigen, Nr. 3

Das Team bietet den Zugehörigen hinsichtlich emotionaler und spiritueller Bedürfnisse, die durch die gezielte Sedierung hervorgerufen werden, Unterstützung an.

Geltungsbereich: SSPV, SAPV.

Bitte geben Sie an, ob Sie der Handlungsempfehlung zustimmen oder diese ablehnen.

- ◯ Zustimmung
- ◯ Ablehnung

Kommentarfeld

72 Standardseite

Thema: Begleitung der Zugehörigen, Nr. 4

Die Zugehörigen werden beraten und ggf. angeleitet, wie sie die Patientin/den Patienten während der gezielten Sedierung unterstützen und ihm/ihr nahe sein können, z.B. durch Reden, Berührung, Schaffen einer für die Patientin/den Patienten wohltuenden Atmosphäre (z.B. Lieblingsmusik, Düfte, Singen bekannter Lieder, Vorlesen, Gebet) sowie - wenn gewünscht - mit in die pflegerische Versorgung (z.B. Mundpflege) einbezogen.

Geltungsbereich: SSPV, SAPV.

Bitte geben Sie an, ob Sie der Handlungsempfehlung zustimmen oder diese ablehnen.

- ○ Zustimmung
- ○ Ablehnung

Kommentarfeld

73 Standardseite

Thema: Begleitung der Zugehörigen, Nr. 5

Vor einer tiefen Sedierung, die bis zum Tod voraussichtlich nicht mehr reduziert wird oder einer Sedierung, die in eine tiefe kontinuierliche Sedierung übergehen kann, ist der Patientin/dem Patienten und den Zugehörigen die Möglichkeit zu geben, sich voneinander zu verabschieden, falls es die Situation zulässt.

Geltungsbereich: SSPV, SAPV.

Bitte geben Sie an, ob Sie der Handlungsempfehlung zustimmen oder diese ablehnen.

○ Zustimmung

○ Ablehnung

Kommentarfeld

74 Standardseite

Thema: Begleitung der Zugehörigen, Nr. 6

Nach dem Versterben der Patientin/des Patienten wird den Zugehörigen die Möglichkeit eines Gesprächs mit Mitgliedern des Behandlungsteams gegeben, um über ggf. verbleibende Bedenken bezüglich der gezielten Sedierung zu sprechen.

Geltungsbereich: SSPV, SAPV.

Bitte geben Sie an, ob Sie der Handlungsempfehlung zustimmen oder diese ablehnen.

○ Zustimmung

○ Ablehnung

Kommentarfeld

75 Standardseite - Unterstützung im Team

Es folgen Handlungsempfehlungen zum Thema: Unterstützung im Team.

76 Standardseite

Thema: Unterstützung im Team, Nr. 1

Alle beteiligten Mitglieder des Teams sollen die Indikation und die Behandlungsziele für die gezielte Sedierung verstehen. Die Diskussion hierzu kann z.B. in Teamsitzungen und Fallkonferenzen stattfinden.

Geltungsbereich: SSPV, SAPV.

Bitte geben Sie an, ob Sie der Handlungsempfehlung zustimmen oder diese ablehnen.

- ○ Zustimmung
- ○ Ablehnung

Kommentarfeld

77 Standardseite

Thema: Unterstützung im Team, Nr. 2

Die Besprechung belastender Situationen im Rahmen der gezielten Sedierung, z.B. in einer retrospektiven Fallbesprechung oder Supervision, wird empfohlen. Ziele solcher Besprechungen sind die Diskussion der sachlichen und der emotionalen Herausforderungen, Hilfe zur Verarbeitung der Belastungen im Team und kontinuierliche Verbesserung der Betreuung.

Geltungsbereich: SSPV, SAPV.

Bitte geben Sie an, ob Sie der Handlungsempfehlung zustimmen oder diese ablehnen.

- ○ Zustimmung
- ○ Ablehnung

Kommentarfeld

78 Standardseite - Abschluss und soziodemografische Daten

Die einzelnen Handlungsempfehlungen sind hiermit alle geschafft!

Es folgt abschließend noch die Möglichkeit zum Geben von Rückmeldungen (via Freitextfeld) sowie die Erfassung von sozio-demografischen Daten.

79 Standardseite

Gleich haben Sie es geschafft! Hier können Sie gerne noch abschließend weitere Rückmeldungen und Anregungen geben.

Auch ist es jetzt ein letztes Mal möglich zuvor gegebene Antworten anzupassen oder Antwortmöglichkeiten zu überarbeiten.

Abschließend folgt nur noch ein kurzer Abschnitt zu Ihren sozio-demografischen Daten.

80 Standardseite

Wie alt sind Sie?

Wie lange sind Sie bereits in dem Bereich der Spezialisierten Palliativversorgung tätig?

Eingabe in Jahren.

Welcher Berufsgruppe bzw. welcher Profession gehören Sie an?

- ☐ Ärztin/Arzt
- ☐ Pflegefachperson
- ☐ Psychologie

- [ ] Physiotherapie
- [ ] Sozialarbeit
- [ ] Weitere

In welchem Bereich der Spezialisierten Palliativversorgung sind Sie tätig?

Eine Mehrfachauswahl ist möglich.

- [ ] Spezialisierte Stationäre Palliativversorgung
- [ ] Spezialisierte Ambulante Palliativversorgung

81 Endseite

Geschafft - vielen herzlichen Dank für Ihre Teilnahme!!

Sie können das Fenster nun schließen.
